# Dlx5 and Dlx6 can antagonize cell division at the G_1_/S checkpoint

**DOI:** 10.1186/s12860-019-0191-6

**Published:** 2019-04-11

**Authors:** Rachel K. MacKenzie, Parvathy Ravi Sankar, Andrew J. Bendall

**Affiliations:** Department of Molecular and Cellular Biology, University of Guelph, 50 Stone Rd East, Guelph, Ontario N1G 2W1 Canada

**Keywords:** Dlx genes, Cell cycle, Checkpoint, Proliferation

## Abstract

**Background:**

*Dlx5* and *Dlx6* stimulate differentiation of diverse progenitors during embryonic development. Their actions as pro-differentiation transcription factors includes the up-regulation of differentiation markers but the extent to which differentiation may also be stimulated by regulation of the cell cycle has not been addressed.

**Results:**

We document that expression of *Dlx5* and *Dlx6* antagonizes cell proliferation in a variety of cell types without inducing apoptosis or promoting cell cycle exit. Rather, a variety of evidence indicates that elevated *Dlx5* and *Dlx6* expression reduces the proportion of cells in S phase and affects the length of the cell cycle.

**Conclusions:**

Antagonism of S-phase entry by Dlx5 and Dlx6 proteins likely represents a lineage-independent function to effect Dlx-mediated differentiation in multiple progenitor cell types.

## Background

Embryonic development unfolds as a series of cell autonomous and extracellular transactions that define each cell’s capacity to divide or differentiate. The rapid cycling of early blastomeres in metazoan embryos gives way to longer and more heavily regulated cell cycles [1] that can respond to differentiation-inducing conditions and generate committed, lineage-restricted, progenitors. Subsequently, terminal differentiation is typically accompanied by a permanent cell cycle exit [2, 3]. The reciprocal relationship between cellular proliferation and differentiation points to a tight coordination between cell cycle dynamics and cell fate, with the time spent in G1 being particularly important. A short G1 phase corresponds to pluripotency in embryonic stem cells [4, 5] and neural precursors undergoing proliferative cell divisions have a shorter G1 phase which lengthens when neurogenic divisions commence [6, 7]. The importance of the time spent in G1 for differentiation has been demonstrated by experimental manipulation of CDK activity; an artificially lengthened G1 phase is sufficient to induce neuronal differentiation [8]. Indeed, a number of cell type-specific transcription factors are known to promote cellular differentiation, at least in part, by directly controlling the expression or function of cell cycle regulators. Classic studies involving the muscle-specific regulatory transcription factor MyoD revealed that its function as a master regulator of myogenesis involved the activation of CDK inhibitors [9, 10], genes that are likely direct physiological targets of the transcription factor [11]. Up-regulation of cell cycle antagonists by differentiation-inducing transcription factors is likely a broadly applicable mode of action, since *p21*^*WAF1/CIP1*^ has also been identified as a target of erythroid-specific factors GATA-1 [12] and EKLF [13]. Alternatively, the down-regulation of cyclin-encoding genes can lead to the same functional outcome; NEUROG2 acts to repress the transcription of various cyclins via direct and indirect means [14]. Prospero does both in *Drosophila* neuroblasts, inhibiting *cyclin E* and the *cdc25* homologue *string* while activating the CDK inhibitor *dacapo* [15, 16]. It should be noted that expression of such differentiation-inducing factors is not incompatible with cell division; rather, mechanisms exist to maintain the proliferative capacity of lineage-committed progenitors. In myogenic precursors, MyoD function is inhibited by the actions of cyclin D1 [17, 18] and NEUROG2 target gene selection is modified by CDK-dependent phosphorylation [19, 20].

Vertebrate *Dlx* genes constitute a family of cell-type specific transcription factors that promote the differentiation of a variety of very different cell types including cortical and olfactory interneurons, chondrocytes, osteoblasts, and ameloblasts, as well as cells in the basal epidermis, and placenta [21–27]. In particular, the co-expressed paralogs *Dlx5* and *Dlx6* are required for the proper maturation and function of cortical [28] and olfactory bulb interneurons [29–32], and sensory cells of the inner ear [33–36], as well as the differentiation of chondrocytes and osteoblasts [35–38]. There is a significant body of evidence to indicate that the pro-differentiation functions of Dlx5 and Dlx6 proteins include their actions as transcriptional activators of lineage-specific genes that define the differentiated cell type [39–43] or of other regulators of lineage-specific differentiation [40, 44–51]. Thus, the differentiation function of Dlx5 is understood on the basis of the activation of lineage-specific markers. In contrast, the effects of Dlx factors on the cell cycle has not been systematically studied. To do so has become increasingly important given numerous observations that elevated *Dlx* gene expression in a variety of solid tumors and hematologic malignancies is compatible with deregulated proliferation [52–56]. To address this deficiency in our understanding of *Dlx* gene function during development we have characterized the effect(s) of Dlx5 and Dlx6 on cell division in a variety of non-tumorigenic cell types. Consistently, we find that expression of these homeodomain regulators antagonizes proliferation without stimulating apoptosis or promoting cell cycle exit. Rather, several lines of evidence points to the G_1_/S transition as a key locus of control.

## Results

### Forced expression of Dlx5 and Dlx6 is sufficient to antagonize cell growth

**Fig. 1:**
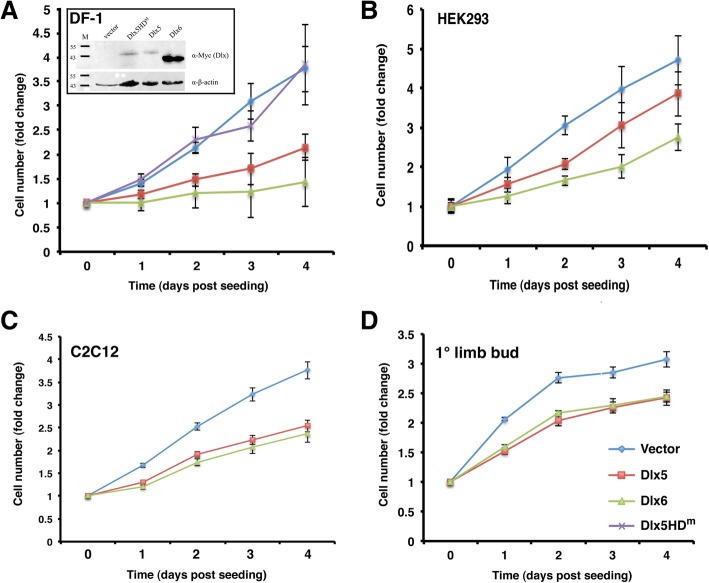
Dlx proteins inhibit growth of a variety of cell types. **a** Transfected and transduced DF-1 cells were seeded in triplicate at 1.2 × 10^4^ cells/well in a 96-well plate. Stable expression of myc-tagged proteins was confirmed by immunoblotting (inset). **b** Transfected and selected HEK293 cells were seeded in triplicate at 1 × 10^4^ cells/well in a 96-well plate without further selection. **c** Transfected and selected C2C12 cells were seeded in triplicate at 1 × 10^4^ cells/well in a 96-well plate without further selection. **d** Chick embryo limb bud cells plus transfected DNA mixture was seeded directly into 96-well plates, in triplicate, at 4 × 10^4^ cells/well. In all panels, relative changes in cell number were measured daily for 4 days using resazurin. All assays were done a minimum of three times for each cell type. Linear regression analysis was performed on the initial increase in cell numbers, and the slopes of the regression lines were compared using the “Comparison of Regression Lines” function in Prism. The slopes of all Dlx5 and Dlx6 regression lines were determined to be significantly different from their respective vector controls

There has been no systematic investigation of the degree to which the up-regulation of *Dlx* gene expression in differentiating tissues impacts the cell cycle or whether there is a specific step in cell cycle progression that is regulated by Dlx proteins. To test the sufficiency of Dlx5 and Dlx6 to antagonize cell division and the generality of this effect we initially tested cell populations that are not known to differentiate in response to endogenous *Dlx* gene expression. We transfected the immortalized chick fibroblast cell line DF-1 with avian retroviral plasmids encoding chicken Dlx5 or Dlx6 and relied on secondary transduction by replication-competent virus in culture to achieve widespread *Dlx* misexpression. Expression of Dlx5 or Dlx6 in DF-1 cells resulted in a much reduced rate of cell accumulation in vitro (Fig. 1a). We also tested whether DNA binding by Dlx5 was required for this effect by expressing a Dlx5 protein (Dlx5HD^m^) with amino acid substitutions in the amino-terminal arm of the homeodomain [57]. DF-1 cells expressing Dlx5HD^m^ grew indistinguishably from DF-1 cells transduced with the empty retrovirus. Thus, the effects of Dlx5 on cell growth in vitro appears to require the DNA binding activity of the homeodomain and, given the very high level of conservation between Dlx homeodomains [22], the same would hold true for Dlx6. We next mis-expressed murine Dlx5 or Dlx6 in the human embryonic kidney epithelial cell line HEK293. The mouse and human Dlx5 and Dlx6 proteins are 97 and 96% identical respectively, permitting the use of this heterologous cell line. Transfected HEK293 cells were selected to enrich for Dlx-expressing cells then cultured without further selection. Again, both Dlx5 and Dlx6 suppressed the rate of cell accumulation over 4 days (Fig. 1b).

We next tested two cell populations that are known to activate expression of *Dlx5* and differentiate in response to developmental signaling cues. The myogenic mesenchymal cell line C2C12 will trans-differentiate into osteoblasts in response to BMP signaling, an effect that is mediated by *Dlx5* [58–61]. Indeed *Dlx5* is sufficient to activate downstream osteogenic regulators in this cell line in the absence of BMP ligand [59]. Transfected C2C12 cells were selected to enrich for *Dlx*-expressing cells then cultured without further selection. As we observed for DF-1 and HEK293 cells, both murine Dlx5 and Dlx6 suppressed the rate of cell accumulation over 4 days (Fig. 1c). Finally, we tested the effects of these genes in primary cells from the early limbs of the chick embryo since *Dlx5* expression is induced in response to chondrogenic signals in the mesenchymal core of the early limb bud [57]. Transiently transfected primary limb bud cells were grown as above and were similarly affected by forced expression of chick *Dlx5* and *Dlx6* (Fig. 1d). For all cell types, the strongest effects were seen in the first 48 h after seeding such that both the rate of cell accumulation and the total cell number at the end of the assay were significantly lower. Since C2C12 cells represent a well studied multipotent progenitor cell model that is responsive to Dlx5-mediated differentiation we focused further analysis on this cell line. For all experiments C2C12 cells were grown under growth-promoting, rather than differentiation-inducing, conditions.

### Dlx-mediated antagonism of cell growth is not the result of increased cell death or cell cycle exit

**Fig. 2:**
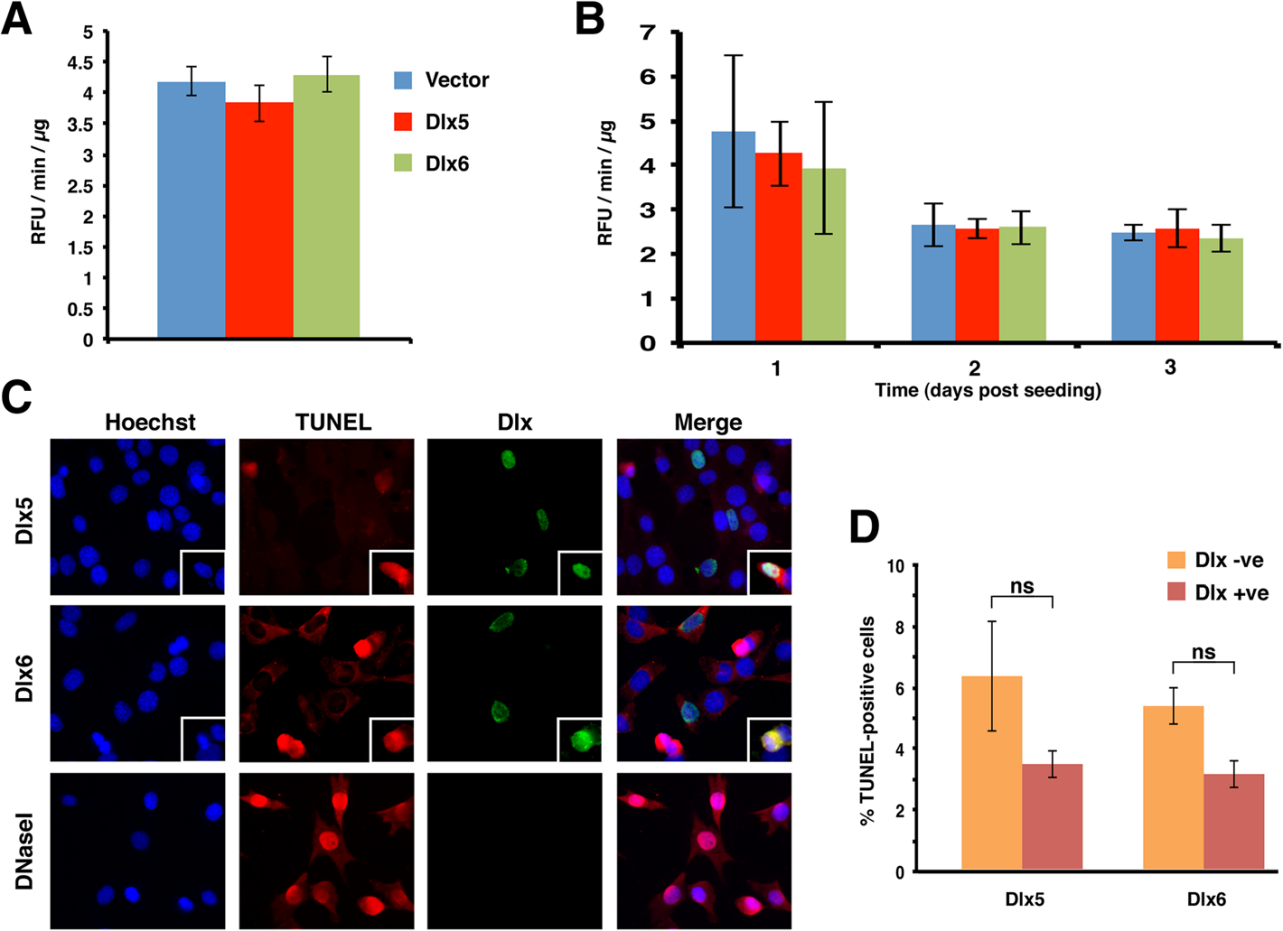
*Dlx*-expressing C2C12 cells do not show elevated levels of apoptosis. **a** At the time of seeding the resazurin viability assay, extra C2C12 cells were collected to assess caspase-3 activation levels. Bars represent the average caspase-3 activity, reported as Relative Fluorescence Units per minute per microgram (RFU/min./μg), +/− the standard error of the mean (SEM). **b** C2C12 cells were transfected and selected in the same manner as for the proliferation assay (*n* = 2). After 4 days of selection, cells were seeded into 6-well plates in triplicate, at a density of 1.9 × 10^5^ cells/well. At 24, 48 and 72 h-post seeding, floating and adherent cells were collected and extracts assayed for caspase-3 activity. Bars represent the average caspase-3 activity (RFU/min./μg) +/− SEM. There was no significant difference in caspase-3 activation in any *Dlx5*- or *Dlx6*-transfected populations compared to the vector control at any time point (ANOVA). **c**, **d** Proliferating C2C12 cells were transiently transfected during seeding onto poly-D-Lysine-coated coverslips and double strand DNA breaks were detected 24 h post-transfection. **(c)** Representative pictures taken with a 40x objective lens. Insets are examples of Dlx-positive and TUNEL-positive cells. **d** The proportion of *Dlx*-expressing cells undergoing apoptosis was compared to the surrounding Dlx-negative population. Bars represent two experiments ± SEM, with at least 100 Dlx-positive cells counted in each. No significant differences in % TUNEL labeling were detected (ANOVA)

To assess the extent to which increased levels of cell death may have contributed to the reduced accumulation of *Dlx5*- or *Dlx6*-expressing cells we measured levels of activated Caspase-3 in the same transfected and selected cells that were used for the growth assay. Levels of activated Caspase-3 in both Dlx5 and Dlx6-expressing cells were indistinguishable from the vector control cells at day zero of the growth assay (Fig. 2a). Thus apoptotic pathways were not activated over control levels during the post-transfection period of G418 selection. To rule out that either Dlx protein induced apoptosis over the course of the growth experiment we seeded similarly-selected cells and measured levels of activated Caspase-3 over 3 days. We recovered detached cells along with the adherent monolayer to ensure a complete recovery of dead and dying cells. Active Caspase-3 activity declined over the course of 3 days and at no time did Dlx-expressing cells have elevated levels of Caspase-3 compared to the empty vector control cells (Fig. 2b). We independently confirmed this observation by TUNEL labeling in C2C12 cells transiently transfected with *Dlx5*- or *Dlx6*-expression plasmids (Fig. 2c, d). In this experiment we could compare Dlx-positive cells to the untransfected cells in the same dish. The proportion of TUNEL-positive cells actually averaged lower in Dlx5- or Dlx6-expressing C2C12 cells (about 3%) compared to co-cultured Dlx-negative cells (5–6%), although the difference did not reach statistical significance (Fig. 2d). Collectively this data supports the interpretation that the reduced cell accumulation we observed in several cell types results from a negative influence on cell division by Dlx5 and Dlx6.

**Fig. 3:**
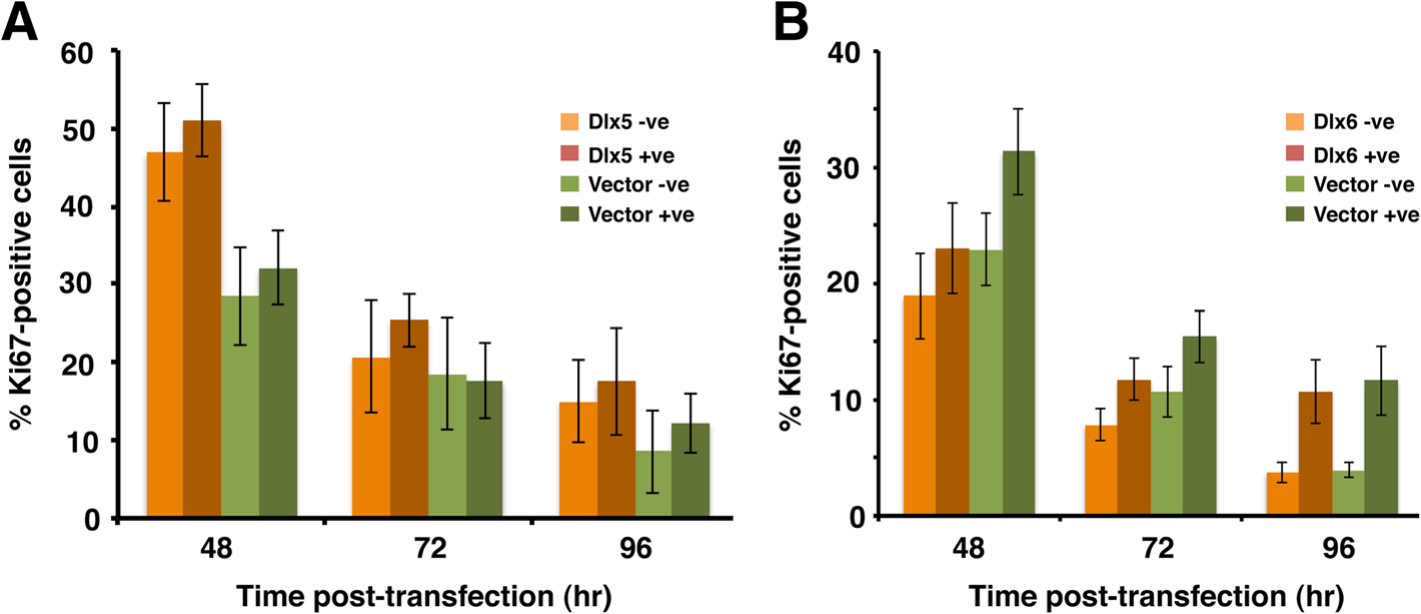
Dlx proteins do not promote cell cycle exit. **a** Proliferating C2C12 cells were co-transfected with a nuclear GFP-encoding plasmid and either a Dlx5-encoding plasmid or an empty vector and seeded onto poly-D-Lysine-coated coverslips. Ki67 and GFP were detected at the times shown post-transfection. The proportion of actively cycling (Ki67-positive) cells was determined for both the transfected and non-transfected populations on each slide. Bars represent the averages from a minimum of three experiments ± SEM, with at least 300 GFP-positive cells counted in total. **b** As for A, with Dlx6-encoding plasmid. No significant difference was detected at any given time point for any two-way comparison (unpaired *t*-tests)

We next asked whether Dlx5 and Dlx6-expressing cells remained in the cell cycle or whether expression of these proteins promoted withdrawal into G_0_. We therefore quantitated the proportion of Ki67-positive cells in Dlx-expressing and control cells. Transiently transfected C2C12 cells were monitored daily between 2 and 4 days post-transfection by co-detection of exogenous transfected protein and endogenous Ki67. The proportion of Ki67-positive cells was quantitated for both transfected and non-transfected cells in the same culture and compared to empty-vector transfected cultures (Fig. 3). As expected, the proportion of Ki67-expressing cells decreased over time as the cultures became confluent and cells entered quiescence. At each time point examined though, the proportion of actively cycling Dlx5-positive cells was indistinguishable from untransfected cells in the same dish and were not statistically different from the empty-vector transfected populations (Fig. 3a). Equivalent results were obtained when the Ki67 status of Dlx6-transfected C2C12 cells was quantitated (Fig. 3b). Thus, neither Dlx5 nor Dlx6 induces quiescence in C2C12 cells cultured under growth-promoting conditions.

### Dlx5 and Dlx6 antagonize entry into S-phase

**Fig. 4:**
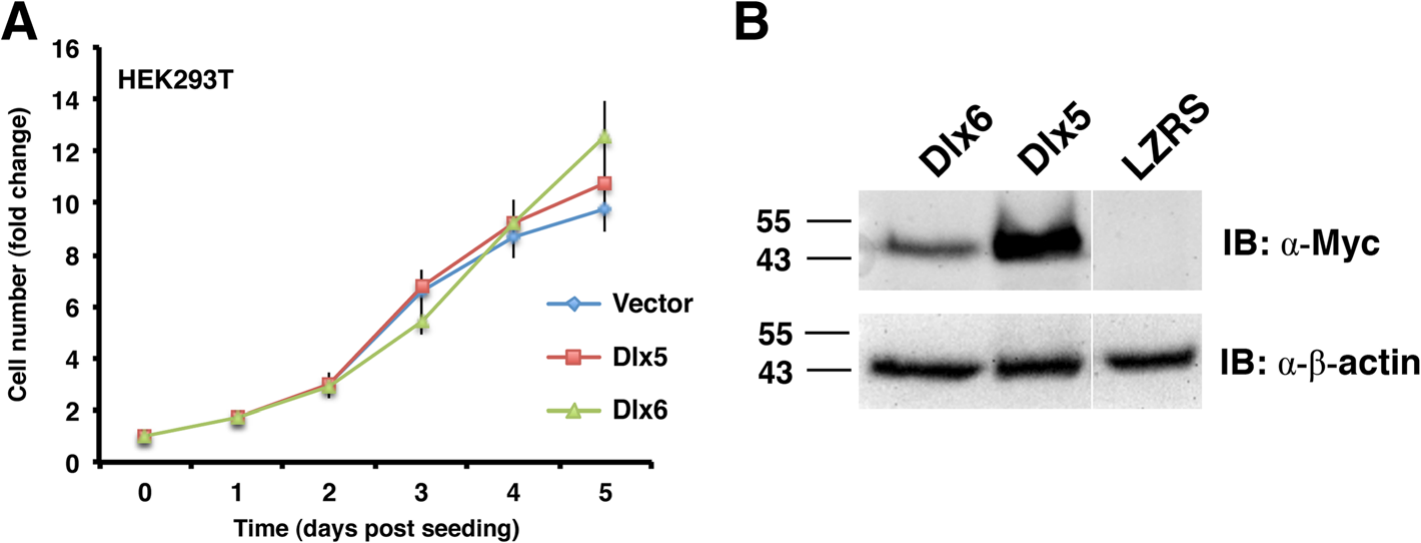
HEK293T cells are refractory to the growth suppressing effects of forced *Dlx* expression. **a** Transfected and selected HEK293T cells were seeded in triplicate at 1 × 10^4^ cells/well in a 96-well plate without further selection. Relative changes in cell number were measured daily for four days with resazurin (*n* = 4). **b** At the time of seeding the growth assay, extra cells were collected for immunoblot (IB) analysis to verify protein expression. Molecular mass standards are shown at left

**Fig. 5:**
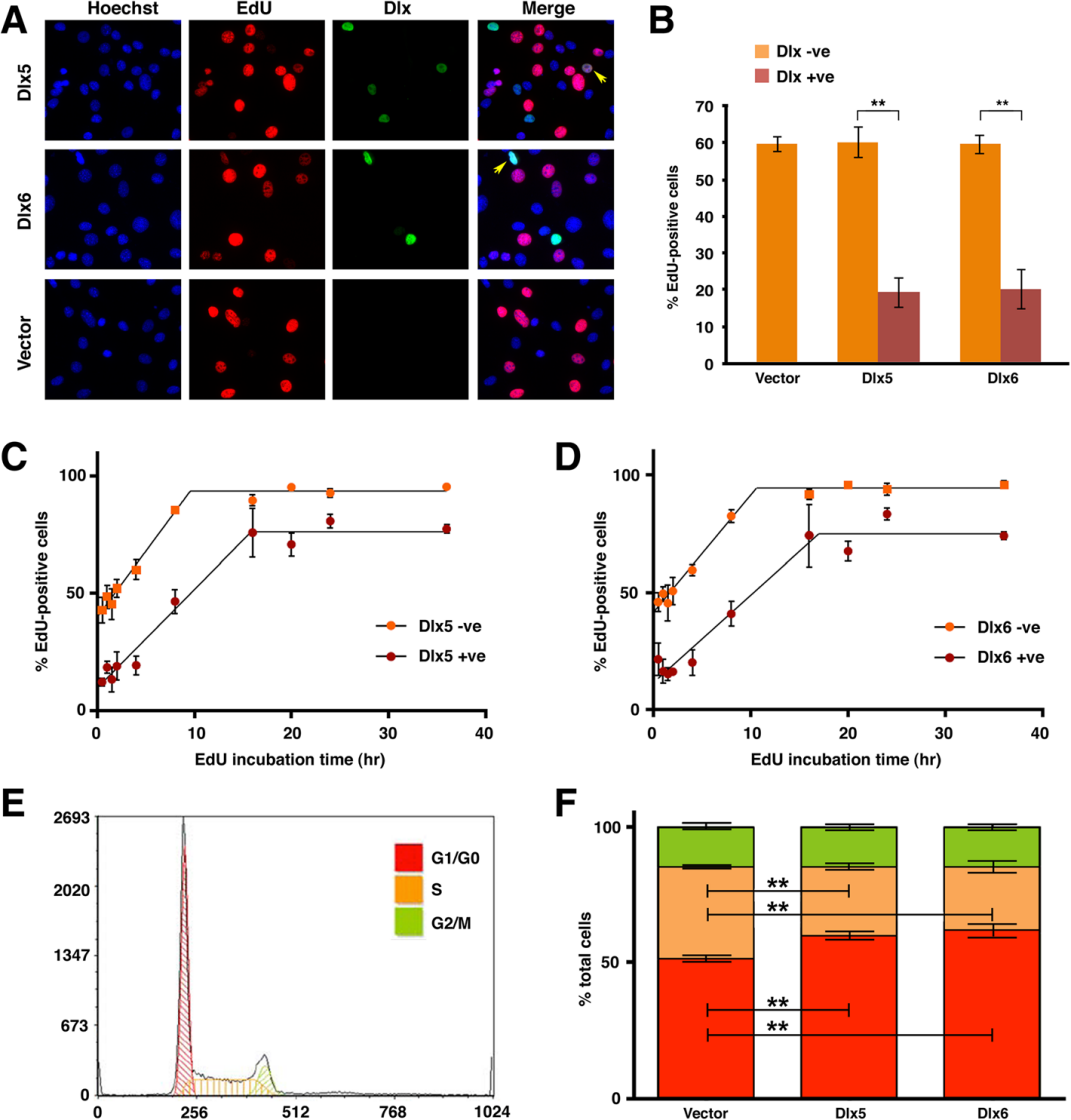
Dlx proteins limit entry into S-phase. **a**, **b** Proliferating C2C12 cells were transfected and seeded onto poly-D-Lysine-coated coverslips and DNA synthesis was detected 24-h post transfection by incorporation of EdU for 4 h. **a** Representative pictures taken with a 40x objective. Yellow arrows point to Dlx-positive cells that have incorporated EdU during the incubation time. **b** Quantitation of the labeling index (LI), defined as the ratio of EdU-positive nuclei to total cells. Bars represent the average LI, +/− SEM from 3 experiments, with at least 200 Dlx-positive cells counted per experiment. **p < 0.01 (unpaired *t*-test). **c**, **d** C2C12 cells, treated as above, were labeled with EdU for various times to measure the growth fraction (GF) and cell cycle length. Each time point represents a minimum of three experiments. LI is plotted over time for (**c**) Dlx5- and (**d**) Dlx6-expressing cells. Slopes of the initial linear increases, as determined by linear regression analysis, were compared in Prism using the “Comparison of Regression Lines” function. Neither Dlx5 nor Dlx6 significantly altered the rate of EdU incorporation, compared to the surrounding Dlx-negative cells (Dlx5 *p* = 0.07; Dlx6 *p* = 0.14). GF was set as the mean of all points following the initial linear increase. The elevations of the GF reached by Dlx-positive and Dlx-negative cells were compared using the “Comparison of Regression Lines” function in Prism, and both proteins were found to significantly alter the GF, compared to the surrounding Dlx-negative cells (Dlx5, Dlx6 *p* < 0.0001). **e**, **f** Transfected C2C12 cells were selected over a period of 4 days then 8 × 10^5^ cells were seeded into 6 cm plates. Cells were collected for flow cytometric analysis and extra cells were re-plated at the original density every 24 h. **e** Representative flow cytometry histogram at 48 h post selection. Scaling on both axes is linear. **f** Quantitation of flow cytometry data. Bars represent the mean of all experiments (*n* = 4) +/− SEM; G1/G0 (red), S (orange), G2/M (green). Both Dlx5- and Dlx6-expressing populations contained a reduced proportion of cells in S-phase, with a compensatory increase in G_1_. **p < 0.0001, unpaired *t*-test

We next wanted to know whether there was a specific point of the cell cycle that was susceptible to Dlx5 or Dlx6 misexpression. In the course of testing the growth effects of Dlx5 and Dlx6 on a variety of cell lines we noted that HEK293T cells were refractory to the growth-inhibitory effects of these proteins (Fig. 4). HEK293T cells differ from the parental HEK293 line in that they express the large T antigen of SV40 [62] and it is known that this viral protein promotes passage through the G_1_/S checkpoint via antagonism and functional subversion of Rb and p53 [63–67]. We therefore focused on this checkpoint and characterized the DNA replication status of Dlx-expressing C2C12 cells. Transfected cells were incubated in the presence of 5-ethynyl-2′-deoxyuridine (EdU), a thymine analogue. Incorporation of EdU into newly synthesized DNA indicates that the cell was in S phase at some point during the labeling period. Comparison of the proportion of EdU-positive cells in the Dlx5 and Dlx6-positive populations to the Dlx-negative cells on the same slide, as well as to the empty vector-transfected population, revealed that significantly fewer Dlx-expressing cells were in S-phase at the time of EdU labeling. Specifically, the labeling index (LI) of *Dlx*-expressing cells was close to 20% compared to 60% for all Dlx-negative populations (Fig. 5a, b).

A reduction in EdU incorporation in Dlx-expressing cells could be due to a reduction in the proportion of actively dividing cells or a lengthening of the cell cycle. In order to distinguish between these possibilities, we used a cumulative labeling method to estimate cell cycle length and the growth fraction (GF), defined as the number of cells in a population that are actively undergoing cell division [68]. EdU was added to the media of transfected cells and incubated for 30 min to 36 h. Cells were collected at each time point, and the LI of Dlx-positive and Dlx-negative cells was determined. The LI of both Dlx-positive and Dlx-negative cell populations increased linearly until the entire growth fraction had been labeled. While *Dlx5* and *Dlx6*-expressing cells reached a GF of 76 and 75% respectively, the Dlx-negative populations surrounding each of them exhibited a GF of 94% (Fig. 5c, d). While the slopes of the linear increase were not determined to be significantly different in the Dlx-positive populations compared to the Dlx-negative populations, the length of time required to reach maximum labeling was increased by approximately 6 h in both the Dlx5- and Dlx6-expressing cells (Table 1).

**Table 1:** Estimation of cell cycle length in C2C12 cells

Population	Growth fraction (%)	Slope of linear increase	Cell cycle length (GF/slope; hr)	Time to LI_max_* (hr)
Dlx5 + ve	76	4.2	18	16
Dlx5 –ve	94	5.6	17	10
Dlx6 + ve	75	3.7	20	17
Dlx6 –ve	94	5.0	19	11

* time to maximum labeling index

Finally, we used flow cytometry analysis to ask whether Dlx proteins were affecting the cell cycle at other checkpoints. Proliferating C2C12 cells were transfected and selected in the same manner as for the alizarin growth assay. After 4 days of selection, each cell population was seeded at equal density into 3 separate plates. 24 h post transfection, one plate was collected for analysis and the other two plates were re-seeded to the starting cell density to ensure that cell crowding did not influence the cell cycle profile. We focused on 48 h post selection when the maximal effect was seen, namely a reduction in the proportion of cells in S phase concomitant with an increase in the proportion of cells in the G1/G0 fraction (Fig. 5e, f). There was no significant change in the size of the G2/M fraction at any time point, arguing that Dlx proteins were not separately limiting entry into G2, or completion of mitosis. Flow cytometry analysis was also done on unselected, transiently transfected, C2C12 populations 24 h post transfection. At 15% transfection efficiency, no effect was seen, but at 30% transfection efficiency there was a 6% increase in the proportion of G1/G0 cells in *Dlx*-transfected populations in comparison to the vector-control population (data not shown). Collectively these data provide an explanation for the growth-inhibitory effects of Dlx5 and Dlx6, namely antagonism of entry into S phase.

## Discussion

The action of *Dlx* genes to promote differentiation in divergent cell lineages and the intimate connections between cellular proliferation and differentiation suggests that interactions with core cell cycle regulators could give a fuller picture of Dlx protein functions during development. In this study we demonstrated that forced expression of *Dlx5* or *Dlx6* is sufficient to antagonize growth in a variety of cell types. In a cell type that undergoes Dlx-mediated differentiation, the effects on cellular proliferation were explained by antagonism of entry into S-phase. The reduced accumulation of Dlx5- or Dlx6-expressing cells could not be explained by elevated levels of apoptosis in the bulk population (Fig. 2). We could also not detect an increased proportion of Dlx-expressing cells in G_0_ by the criteria of Ki67 levels (Fig. 3), leading us to examine cell cycle kinetics. While the reduced growth fraction in Dlx-expressing cells (Fig. 5c, d) might otherwise be taken as evidence of a subpopulation of cells in G_0_, the continued expression of Ki67 in Dlx-expressing cells suggests instead that a fraction of the Dlx-expressing cells are not completing their cell cycle over a 36 h period. This latter effect may be an artifact of very high protein levels in affected cells where the block to S-phase becomes “permanent”.

We note that their known effects on differentiation make loss-of-function experiments involving *Dlx* genes difficult to interpret when it comes to their roles in regulating proliferation. Preventing *Dlx* expression all together, as has been done in multiple knockout mouse models, delays differentiation in Dlx-dependent cell types but doesn’t result in an obvious overgrowth phenotype [34, 38, 69–72]. Conversely, knocking down *Dlx* expression later in development is not expected to revert cells to a proliferating progenitor state. Exploration of the mechanisms of action of Dlx proteins at the G_1_/S checkpoint will therefore largely depend on gain-of-function experiments until more mechanistic details emerge and more targeted loss-of-function assays can be done.

It some embryonic cellular contexts, expression of *Dlx5* and *Dlx6* is both compatible with proliferation and required for continued tissue expansion. For example, limb bud mesenchyme continues to divide normally in the absence of both *Dlx5* and *Dlx6* function but proliferation in the adjacent AER does not [38]. A similar situation exists in the otic vesicle [34, 38]. Notably, these affected tissues do not differentiate under the influence of *Dlx* gene expression but, rather, act as transient signaling centers for growth and patterning. While cell division can be compromised in such cells by the absence of Dlx5 and Dlx6, elevated expression of either Dlx protein appears to antagonize cell division. In cells that normally express these factors then, it is the *levels* of Dlx5 and Dlx6 that appear to be important for maintenance of cellular proliferation at wild type rates during development: too little Dlx5 and Dlx6 and cell division can be compromised in some cell types, leading to cell death [34]; too much Dlx5 (and presumably Dlx6) and cell division is antagonized, thereby promoting precocious differentiation [57, 73, 74]. While we do not know that elevated levels of Dlx5 or Dlx6 delay entry into S-phase in all progenitor cell types in which they are expressed, our data in a variety of mesenchymal and epithelial cells suggests this is a likely to be a general phenomenon. A number of studies, furthermore, lead us to conclude that it is the collective functional pool of co-expressed Dlx5 and Dlx6 proteins that is the key factor [69, 75]. Many of the functions of these two proteins are interchangeable; morphological and differentiation defects in *Dlx5/6*^*−/−*^ mice are rescued by the tissue-specific expression of a single paralog [37, 38] and, in a number of promoter contexts, these two proteins behave in a quantitatively indistinguishable manner [76].

Since the observation that an increase in cell cycle length accompanies the switch from proliferative to neurogenic cell divisions [6] and that a lengthened G_1_ phase is causative for neurogenesis, rather than a consequence [7, 8], several studies have revealed a mechanistic link between cyclins, cyclin-dependent kinases, CDK inhibitors, and neurogenic differentiation in the central nervous system [77–79]. In neural progenitors, at least, the time spent in G_1_ appears to be the critical variable while S, G_2_, and M phases proceed on a more invariant schedule [6, 80–82]. Elongation in the length of the cell cycle by antagonism of entry into S phase and the coincident accumulation of differentiation-promoting determinants in a lengthened G_1_ is an attractive model [8] that may well apply to other tissues. Indeed, molecular mechanisms that link cell cycle progression to differentiation in other tissues have been identified [3, 83–87]. Dlx action in this study was consistent with such a model since the effect of expression was to prolong the time spent in G_1_, rather than promoting exit to G_0_.

Of specific interest, links between Dlx-mediated jaw morphogenesis and cell cycle regulation warrant investigation. Quail-chick chimera studies have revealed the extent to which the neural crest mesenchyme acts as a time-keeper to influence beak size by controlling timing of the transition from neural crest progenitor proliferation to osteoblast differentiation [88]. Mechanistically, the link appears to involve regulation of cyclin D levels, since manipulation of *ccnd1* expression prematurely up-regulates osteogenic differentiation markers like *Runx2* and results in a smaller beak [88]. Additionally, ventral-specific *edn1*-mediated proliferation of neural crest-derived cells is known to be required for expansion and outgrowth of the jaw, and is counterbalanced by *hand2* [89], a target of *Dlx5* and *Dlx6* [49, 90]. Thus, Dlx5 and Dlx6 likely regulate differentiation of jaw-forming tissues, at least in part, through antagonism of cell cycle progression. Notably, the cell cycle regulators encoded by *dach1*, *tcf19*, and *ccnd2* are all deregulated in the first pharyngeal arch of *Dlx5/6* null E10.5 mouse embryos (microarray data published in [91]). In the chick embryo, *ccnd2* has been explicitly linked to the coordination of proliferation, differentiation and patterning in discrete progenitor domains in the early chick spinal cord [92]. *Ebf1*, a gene that has also been implicated in the coordinated regulation of proliferation, survival, and differentiation [93], is a target gene of Dlx5 and/or Dlx6, in multiple tissue contexts [51, 94]. While such circumstantial evidence places Dlx proteins as DNA-binding transcriptional regulators of cell cycle control genes, the extent to which Dlx-mediated regulation of cell cycle progression involves protein-protein interactions with other cell cycle regulators also remains to be investigated.

## Conclusions

The *Dlx* family of homeobox genes are heavily studied for their important functions in patterning and differentiation in vertebrate embryos. While a large body of literature has documented their roles in promoting differentiation of various cell types through the up-regulation of differentiation markers, the potential of *Dlx* genes to promote differentiation by antagonizing the cell cycle has not been addressed. Here, we document a generalized antagonism of cellular proliferation when *Dlx5* or *Dlx6* are over-expressed. Elevated levels of the proteins did not lead to apoptosis or cell cycle exit. Rather, we have revealed a reduced capacity of *Dlx5*- and *Dlx6*-expressing cells to enter S-phase as a specific consequence of elevated levels of Dlx5 and Dlx6 proteins. Our finding that Dlx proteins influence progression through the G_1_/S checkpoint prompts further work to link *Dlx* genes, morphogenesis, differentiation, and cell cycle progression.

## Methods

### Plasmids

*RCASBP(A)* plasmids encoding N-terminal myc-tagged chick *Dlx5*, N-terminal myc-tagged chick *Dlx6*, or N-terminal myc-tagged mouse *Dlx5HD*^*m*^, as well as *pcDNA3* plasmids encoding N-terminal myc-tagged murine *Dlx5* and N-terminal myc-tagged murine *Dlx6* have been described and used previously [95]. *LZRS-mycDlx6* was made as described for *LZRS-mycDlx5* [57].

### Embryos

Fertile Barred Rock chicken eggs were obtained from the Arkell Poultry barn at the University of Guelph (Guelph, ON), a Canadian Council on Animal Care-certified institution, and incubated at 38 °C and high humidity for 4 days. Work with early stage chick embryos is not reviewed by the University of Guelph Animal Care Committee, according to Canadian Council on Animal Care guidelines. Embryos were staged according to Hamburger and Hamilton. Limb buds of stage 22–24 embryos were dissected in Puck’s Saline G (PSG) [96]. Tissue was digested with 0.25% Trypsin in Calcium- & Magnesium-free PSG plus 10% fetal calf serum. Digested tissue was triturated with a flame-burnished Pasteur pipette until a single cell suspension was observed microscopically and complete media was added. Cells were counted, centrifuged at 900 xg for 5 min. and resuspended at 2 × 10^7^ cells/ml.

### Cell lines, transfection, and selection

DF-1 (ATCC CRL-12203), HEK-293 (ATCC CRL-1573), HEK-293 T (ATCC CRL-3216), C2C12 (ATCC CRL-1772), and primary chick embryo limb bud cells were cultured at 37 °C and 5% CO_2_ in Dulbecco’s Modified Eagle’s Medium (DMEM) supplemented with 10% (or 20% for C2C12) fetal bovine serum, 100 U/ml penicillin, 100 μg/ml streptomycin, and 2 mM L-glutamine. In some experiments, cells were cultured on glass coverslips that had been coated with 0.1 M poly-D-lysine for 30 min. then washed twice with sterile deionized water and once with DMEM. DF-1 cells were transfected at sub-confluency in a 6 cm dish with 4 μg *RCASBP(A)* plasmids using 4.5 μg polyethylenimine (PEI) per μg of plasmid DNA. Cells were expanded to 10 cm dishes 24 h post transfection. In order to enrich for Dlx-expressing cells we relied on the ability of the replication-competent virus made by transfected cells to transduce untransfected cells in the dish. Thus, transfected populations were expanded, after a further 48 h, to 15 cm dishes along with one quarter of a sub-confluent (untransfected) 10 cm dish, the virus-containing media from the transfected cells, and 10 ml of fresh complete DMEM. Cells were collected 48 h later for growth assays. HEK293, and HEK293T cells were transfected at sub-confluency in 6 cm dishes with 4 μg of *LZRS* plasmids using PEI as above. Cells were maintained at sub-confluency and selected with 4 μg/ml Puromycin (Fisher Scientific) for 3–7 days to enrich for Dlx-expressing cells. C2C12 cells were transfected in suspension with 4 μg *pcDNA3* plasmids, using 6 μl Effectene Transfection Reagent (Qiagen) per μg plasmid DNA and EC buffer supplemented with 0.4 M trehalose. Cells were maintained at sub-confluency and selected over 4 days with 1 mg/ml G418 (BioShop). 1.6 × 10^6^ chick embryo limb bud cells, at a density of 2 × 10^7^ cells/ml, were transfected in suspension with 3 μg of *RCASBP(A)* plasmids and Effectene, as above. The DNA/cell mixture was seeded directly into the cell viability assay.

### Resazurin cell viability assay

Cell populations were seeded into 96-well plates at equal density in 100 μl of supplemented DMEM. Each day, 100 μl of 0.05 mM resazurin (Sigma) in supplemented DMEM was added per well. Fluorescence was measured at Ex516λ and Em590λ in a microplate fluorescence reader immediately after resazurin addition and after 4 h of incubation at 37 °C and 5% CO_2_ to aquire a measure of live cells. Measurements on new cells were repeated every 24 h for 5 days. Background-subtracted changes in fluorescence were normalized against the reading taken at day zero.

### Caspase-3 activation assay

To avoid any bias in measurements of caspase-3 activity, floating cells were collected prior to trypsinizing the adherent monolayer at each time point. Cells were incubated for 30 min. on ice in cell lysis buffer (50 mM HEPES, pH 7.4; 0.1% CHAPS; 0.1 mM EDTA; 1 mM DTT) at a density of 1.5 × 10^6^ cells/50 μl. Extracts were centrifuged at 20,000 xg for 10 min. at 4 °C. Caspase activity was measured by mixing 25 μl of cell extract with 75 μl of reaction buffer (50 mM HEPES, pH 7.4; 100 mM NaCl; 0.1% CHAPS; 10 mM DTT; 1 mM EDTA; 10% glycerol) and 100 μl of 60 μM fluorogenic caspase-3 substrate (Ac-DEVD-AMC). The amount of AMC released was measured at 37 °C every 5 min. for 90 min. using a BIO-TEK Flx800 microplate fluorescence reader with 360/40 nm Fluorescence Filter (BIO-TEK) for excitation and 460/40 nm Fluorescence Filter (BIO-TEK) for emission. Protein concentration was determined using BCA protein assay kit (Pierce) at 454 nm, allowing the relative fluorescence units released per minute, per μg of protein to be determined.

### Immunoblotting

Cells were collected in chilled Dulbecco’s Phosphate Buffered Saline (Hyclone) and lysed with sonication in high salt lysis buffer (50 mM Tris, pH 8; 500 mM NaCl; 1% Triton X-100) with protease inhibitors (Complete Mini Protease Inhibitor; Roche). Protein was quantified and separated by SDS-PAGE in a 13% poly-acrylamide gel then transferred to PVDF. Membranes were blocked for at least 1 h in 5% skim milk powder in PBS containing 0.1% Tween-20. Membranes were incubated for at least 2 h in primary antibodies: α-MYC (1:500 monoclonal 9E10 in blocking solution) or anti-β-actin (Genetex at 1:2000) and 1 h in secondary (1:10,000 HRP-conjugated goat-α-mouse, Bio-Rad). Immunoreactive bands were visualized with Western Lightning Chemiluminescence Reagent Plus (PerkinElmer Life Sciences) and imaged with a Bio-Rad molecular Imager Chemi-Doc XRS+ and ImageLab software (Bio-Rad).

### TUNEL assay

Cells were transfected in suspension directly onto poly-D-lysine-coated coverslips and collected 24 h post transfection. Cells were fixed in 3.7% formaldehyde in PBS and permeabilized using 0.25% Triton-X-100 in PBS. An untransfected slide was treated with DNase I to determine the level of fluorescence above background at which to count cells as positive for extensive DNA damage. DNA breaks were detected with a Click-iT® TUNEL Alexa Fluor® 594 Imaging Assay (Life Technologies).

### Indirect immunofluorescence

Following Click-iT® chemistry, coverslips were washed twice in PBS supplemented with 10 mM glycine (PBS-G). Myc-tagged Dlx proteins were detected with 9E10 mouse monoclonal antibody diluted 1:50 with 3% bovine serum albumin (BSA) in PBS-G for 2 h at room temperature in the dark. Coverslips were washed twice with PBS-G then incubated for 45 min. with secondary FITC-conjugated goat anti-mouse antibody (Genetex) diluted 1:100 with 3% BSA in PBS-G. Coverslips were then washed 3 times with PBS-G and incubated for 30 min. in 5 μg/ml Hoechst 33342 (Molecular Probes) at room temperature in the dark. Coverslips were washed twice with PBS and once with sterile deionized water and allowed to dry before mounting in 1,4–19 diazabicyclo [2.2.2] octane (DABCO at 10 mg/ml in 1:9 PBS: Glycerol; 0.02% sodium azide). Ki67 was detected with the same protocol using purified mouse anti-Ki67 monoclonal (BD Pharmingen #550609) at 1:500 dilution in PBS-G + 3% BSA for 2 h and donkey anti-mouse secondary conjugated to Alexa594 (Life Technologies) at 1:500 dilution in PBS-G + 3% BSA for 45 min. Due to antibody incompatibility, Dlx proteins were detected by proxy from co-transfected plasmid encoding nuclear GFP.

### EdU incorporation

Cells were transfected in suspension directly onto poly-D-lysine-coated coverslips and cultured for 24 h before addition of EdU. Transfected cells were incubated for various times with 10 μM EdU in DMEM at 37 °C, 5% CO2, then fixed in 3.7% formaldehyde in PBS and permeabilized using 0.5% Triton-X-100 in PBS. EdU was detected with a Click-iT® EdU Alexa Fluor® 594 Imaging Kit (Life Technologies) according to the manufacturer’s instructions. Indirect immunodetection of epitope-tagged proteins was performed, as above, after EdU detection.

### Flow cytometry

Transfected cells were trypsinized and counted 24 h post seeding. Cells were centrifuged at 900 xg and resuspended at 1 × 10^6^ cells/ml in Dulbecco’s PBS. Cells were centrifuged again at 900 xg and fixed with chilled (− 20 °C) 70% ethanol with vortexing. Cells were then incubated at − 20 °C for at least 24 h then pelleted at 3000 xg. Cell pellets were washed twice with PBS then resuspended in 50 μl of 100 μg/ml RNase A (per 1 × 10^6^ cells) and incubated at room temperature for 10 min. 400 μl of 50 μg/ml propidium iodide in PBS was added (per 1 × 10^6^ cells) and incubated for 30 min. at 37 °C. DNA content, represented as propidium iodide fluorescence, was quantitated using a Cytomics FC 500MPL flow cytometer (Beckman Coulter) at excitation of 488 nm. FL1 (Em 525) was used to detect FITC fluorescence and FL3 (Em 610) was used to detect propidium iodide. FL3 histograms were analyzed using the MultiCycle AV DNA analysis software (Phoenix Flow Systems) available in the FCS Express 4 Plus-Research Edition program (De Novo Software). The histogram was fit with the SL G21 S0 one cycle fitting model. G2/G1 ratio was set at 1.93 and background was removed before G1, S and G2 DNA content was analyzed.

### Statistical analysis

All statistical analysis was performed using Prism (GraphPad). All linear increases in growth curves and cumulative labeling EdU incorporation assays were fit with a linear regression model, considering each replicate Y value as an individual point. The slopes of any 2 lines were compared with a two-tailed test of the null hypothesis that the slopes were identical. A *p* value less than or equal to 0.05 was interpreted as a rejection of the null hypothesis. If the p value was greater than 0.05, the intercepts of the slopes were compared. A second p value was calculated testing the null hypothesis that the lines were identical. A p value of less than or equal to 0.05 was interpreted as a rejection of the null hypothesis. Two-way analysis of variance (ANOVA) was performed on any data that had multiple variables affecting the outcome, including the time-point caspase-3 activation assay, 4 h EdU incorporation assay and TUNEL. Other caspase-3 activation assays were analyzed using a one-way ANOVA. Flow cytometry data was compared with unpaired *t-*tests, using the Holm-Sidak method with an alpha of 5% to correct for multiple comparisons. In all of these instances, a p value less than or equal to 0.05 was considered statistically significant.

## Abbreviations

ANOVA: Analysis of variance
BSA: Bovine serum albumin
DABCO: 1,4–19 diazabicyclo [2.2.2] octane
DMEM: Delbecco’s Modifed Eagle’s Medium
EdU: 5-ethynyl-2′-deoxyuridine
FITC: Fluorescein isothiocyanate
GF: Growth fraction
LI: Labeling index
PBS: Phosphate-buffered saline
PBS-G: Phosphate-buffered saline containing glycine
PEI: Polyethylenimine
PSG: Puck’s saline with glucose
RFU: Relative fluorescence unit
SEM: Standard error of the mean
TUNEL: Terminal deoxynucleotidyl transferase dUTP nick end labeling
